# Prognostic significance of pretreatment controlling nutritional status score in urological cancers: a systematic review and meta‐analysis

**DOI:** 10.1186/s12935-021-01813-2

**Published:** 2021-02-19

**Authors:** Xinhao Niu, Zhe Zhu, Juan Bao

**Affiliations:** Department of Urinary Surgery, Shanghai Public Health Clinical Center, Fudan University, No. 2901 Caolang Road, 201508 Shanghai, China

**Keywords:** CONUT score, Urological cancers, Meta‐analysis, Prognosis

## Abstract

**Background:**

Controlling Nutritional Status (CONUT) score is a novel nutrition-based biomarker that has been reported for predicting survival in various cancers. However, the relationship between CONUT score and prognosis of urological cancers remains unclear. Hence, we performed this meta-analysis to evaluate the prognostic significance of CONUT score for patients with urological cancers.

**Methods:**

PubMed, Embase, the Cochrane Library and National Knowledge Infrastructure (CNKI) were systematically searched up to October 2020. The pooled hazard ratios (HRs) with 95% confidence intervals (CIs) were calculated to evaluate the association of CONUT score with overall survival (OS), cancer-specific survival (CSS) and recurrence/disease/progress-free survival (RFS/DFS/PFS) in urological cancers.

**Results:**

A total of 12 articles with 13 studies were included in the analysis. Pooled results indicated that increased CONUT score predicted poor OS (HR: 1.78, 95% CI 1.51–2.09, p < 0.001), CSS (HR: 2.14, 95% CI 1.55–2.97, p < 0.001) and RFS/DFS/PFS (HR: 1.57, 95% CI 1.35–1.84, p < 0.001). Subgroup analysis by cancer type revealed that high CONUT score associated with worse OS in renal cell carcinoma (RCC) and urothelial cancer (UC) (HR: 3.05, 95% CI 2.07–4.50, p < 0.001; HR: 1.58, 95% CI 1.32–1.89, p < 0.001). Similar results could be found in CSS (RCC HR: 2.67, 95% CI 1.87–3.81, p < 0.011; UC HR: 1.68, 95% CI 1.09–2.59, p = 0.011) and in RFS/DFS/PFS (RCC HR: 1.96, 95% CI 1.44–2.66, p < 0.001; UC HR: 1.42, 95% CI 1.18–1.71, p < 0.001).

**Conclusions:**

These results illustrated that the high CONUT score may predict worse survival for patients suffering from urological cancers. Therefore, the CONUT score may represent an effective prognostic indicator in urological cancers.

## Background

Urological cancers, mostly containing renal cell carcinoma (RCC), prostate cancer (PC) and urothelial cancer (UC), are the major public health problem around the world [1]. According to global cancer statistics, urological cancers accounted for greater than 32% of all kinds of malignant tumors in 2019 [2]. RCC is a common malignant tumor of the urinary system, with more than 70,000 new cases and 10,000 deaths in 2020 [3]. During the latest cancer statistics, PC is predicted to be the third most commonly diagnosed cancer in the world, with more than one million new cases annually [3]. UC, mainly comprising bladder cancer (BC) and upper tract urinary cancer (UTUC), is estimated to cause over 18,000 deaths in the United States each year [4]. Despite the progress of the therapies and techniques for urological cancers including chemotherapy and molecular targeted therapy, the clinical prognosis of urological cancers remains not significantly increase in the past two decades, partly due to recurrence and metastasis [5]. Based on the expected survival time of patients to develop treatment plan are important for improving the cure rate of urological cancers. At present, the treatment of urological cancers is mainly based on pathological stage. Nevertheless, the current stage system is not enough to support the choice of treatment and the evaluation of prognosis of urological cancers [6]. Therefore, it is critical to explore a new prognostic biomarker to guide the treatment of urological cancers.

Accumulating evidence demonstrates that host nutritional status plays an important role in progression of cancers and survival of cancer patients [7]. Several nutritional assessment biomarkers such as prognostic nutritional index (PNI) [8], Glasgow prognostic score (GPS) [9], modified Glasgow prognostic score (mGPS) [9], albumin-to-globulin ratio (AGR) [10] and serum albumin [11] have been confirmed as prognostic factors in urological cancers. The CONUT score, calculated from three peripheral blood parameters (total lymphocyte count, serum albumin concentration and total cholesterol level, Table 1), is a newly proposed scoring system to evaluate nutritional status of patients. The three peripheral blood parameters were convenient blood parameters and easy to be acquired during routinely clinical practice. Furthermore, compared to the biomarkers (PNI, GPS, mGPS, AGR and serum albumin) which were determined from only one or two types of serum markers, the CONUT score may be able to provide a more comprehensive clinical picture of the balance of host nutritional and immune status since it is derived from up to three blood parameters. High CONUT signifies low levels of lymphocytes, albumin and cholesterol, which is typically associated with the poor nutritional and immune status of patients and it can lead to worse survival. Recently, the prognostic value of the CONUT score has been reported in gastric cancer [12], hepatocellular carcinoma [13] and colorectal cancer [14]. Nonetheless, the application of CONUT score as a prognostic indicator in patients with urological cancers remains inconsistent. For example, Miyaka et al. [15] and Takemura et al. [16] considered high CONUT score was not associated with patients’ prognosis, while other studies [17, 18] suggested CONUT score was an effective prognostic indicator in urological cancers. Thus, we aimed to systematical review the published literatures, and investigate CONUT score as prognosis predictor in urological cancers, so as to provide high-level evidence for this issue.

**Table 1:** Definition of CONUT score

Parameters	CONUT			
	Normal	Light	Moderate	Severe
Serum albumin (g/dL)	3.5–4.5	3.0-3.49	2.5–2.99	< 2.5
Sore	1	2	4	6
Total lymphocyte (count/mm^3^)	≥ 1600	1200–1599	800–1199	< 800
Sore	0	1	2	3
Total cholesterol (mg/dL)	> 180	140–180	100–139	< 100
Sore	0	1	2	3
CONUT score (total)	0–1	2–4	5–8	9–12

## Materials and methods

### Search strategy and selection criteria

This work was conducted according to the preferred reporting items for systemic reviews and meta-analysis (PRISMA) [19]. A comprehensive literature search for relevant studies to assess the relationship between the CONUT score and prognosis of urological cancers in PubMed, Embase, the Cochrane Library and National Knowledge Infrastructure (CNKI) databases, published up to October 2020. The used search terms were “controlling nutritional status”, “CONUT”, “renal cell cancer”, “bladder cancer”, “prostate cancer”, “urothelial cancer”, “urological cancer”, “prognosis”, and “survival”.

Two independent authors (XHN and ZZ) reviewed all candidate articles. Literatures were finally included when they met the following criteria: (1) patients were histopathologically diagnosed with urological cancers, including RCC, BC, PC UTUC; (2) reported the relationship between pretreatment CONUT score and overall survival (OS) or cancer-specific survival (CSS) or recurrence/disease/progress-free survival (RFS/DFS/PFS); (3) provided hazard ratio (HR) and 95% confidence intervals (CI) directly. Studies were excluded when they met the following criteria: (1) letters, reviews, abstract and case reports; (2) lack of accurate data; (3) the cut-off value did not given.

### Data extraction and quality assessment

The following data were extracted by two authors (XHN and ZZ): first author’s surname, country of origin, year of publication, number of patients, duration of follow-up, cancer type and stage, treatment methods, design of study, cut-off value, and survival analysis. The quality of all primary studies were independently assessed in the light of the Newcastle-Ottawa Scale (NOS) [20] by three investigators (XHN, ZZ and JB). Total quality scores were ranged from 0 to 9 and if the final score > 6, we regarded it to be of high quality.

### Statistical analysis

HR and 95% CI were directly extracted from each publication to evaluate the importance of prognostic role of CONUT score for patients with urological cancers. To pool the overall HR with 95% CI, the HR from the multivariate analysis was extracted from each study. The Cochran Q and I^2^ statistical methods were applied to evaluate the heterogeneity among included studies [21]. A fixed-effects model was used to calculate the pooled estimates in the absence of heterogeneity (I^2^ < 50% or p > 0.10). Otherwise, a random-effects model was applied. According to cancer type, cancer stage, treatment methods, sample size and cut-off value, further subgroup analysis was also conducted. Potential publication bias was evaluated by Begg’s test with funnel plots. A p-value < 0.05 was regarded statistically significant. In addition, a sensitivity analysis was carried out to assess the influence of each individual study on the pooled results by sequentially excluding each study. All analyses were performed by using Stata software version 12.0.

## Results

### Characteristics of selected articles

**Fig. 1:**
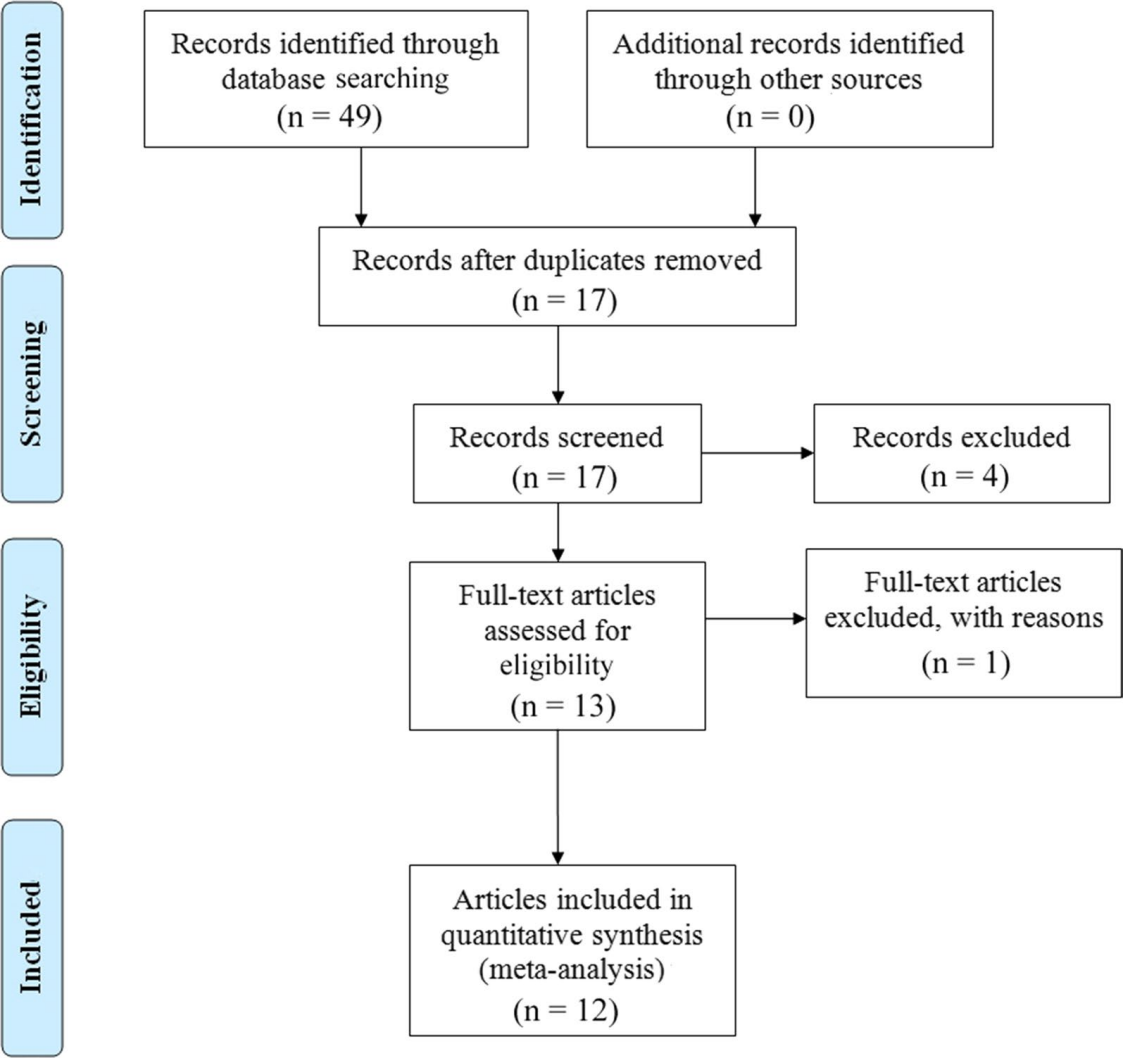
Flow chart of the meta-analysis

The selection procedure was presented in a flow diagram (Fig. 1). Finally, 12 articles with 13 studies were included for meta-analysis [15–18, 22–29]. The features of the 13 studies were outlined in Table 2. All included studies were evaluated as high quality according to the NOS (Table 3). Among the studies, 6 focused on RCC, 6 focused on UC and 1 focused on PC. Of six studies on UC, 2 investigated bladder urothelial cancer and 3 investigated UTUC. A total of 6 studies were conducted in China, 4 performed in Japan, 2 from Korean and 1 from Egypt. The sample sizes were ranged from 49 to 1418. In terms of treatment methods, target therapy was investigated in 1 studies and operation was investigated in 11 studies. Among the included studies, 9 evaluated OS, 9 evaluated CSS, 8 evaluated RFS/DFS/PFS.

**Table 2:** Characteristics of studies included in this meta-analysis

Study, year	Country	Duration	Sample size	Median follow-up (months)	cancer type	Stage	Treatment	Study design	Survival outcome	Cut-off	NOS
Guo 2017	China	2010–2011	189	45	BC	Mixed	Surgery	R	OS	3	7
Miyake 2017	Japan	2006–2016	117	NR	BC	Non-metastatic	Surgery	R	OS,CSS	1	8
Zhang 2019	Chian	2015–2017	94	16.31	PC	Metastatic	Surgery	R	PFS	3	7
Elghiaty 2019	Egypt	2005–2014	1046	63	RCC	Non-metastatic	Surgery	R	OS,CSS,RFS	2	8
Kang 2018 1	Korean	1999–2015	1418	41	RCC	Mixed	Surgery	R	CSS	2	7
Kang 2018 2	Korean	1999–2015	1282	41	RCC	Non-metastatic	Surgery	R	RFS	2	7
Song 2019	China	2010–2012	325	64	RCC	Non-metastatic	Surgery	R	OS,CSS,DFS	3	8
Takemura 2020	Japan	2016–2019	49	26.4	RCC	Metastatic	Target therapy	R	CSS,PFS	2	7
Zheng 2018	China	2004–2014	635	48.4	RCC	Non-metastatic	Surgery	R	OS,CSS	2	8
Ishihara 2017	Japan	2003–2014	107	Mean: 41.48	UTUC	Non-metastatic	Surgery	R	OS,CSS,RFS	3	7
Xu 2018	China	2004–2016	572	41	UTUC	Mixed	Surgery	R	OS,CSS,RFS	2	8
Bao 2020	China	1999–2015	408	32	UTUC	Non-metastatic	Surgery	R	OS,CSS,DFS	3	8
Suzuki 2020	Japan	2003–2018	163	12.3	UC	Metastatic	Mixed	R	OS	2	8

*BC* bladder cancer, *PC* prostate cancer, *RCC* renal cell carcinoma, *UTUC* upper tract urinary cancer, *UC* urothelial cancer, *NR* not reported, *R* retrospective, *OS* overall survival, *CSS* cancer-specific survival, *DFS* disease-free survival, *RFS* recurrence-free survival, *PFS* progress-free survival, *NOS* Newcastle-Ottawa Scale

**Table 3:** Newcastle–Ottawa Scale for quality assessment

Studies	Quality indicators from Newcastle–Ottawa Scale								Scores
	1	2	3	4	5	6	7	8	
Guo 2017	★	★	–	★	★★	★	★	–	7
Miyake 2017	★	★	–	★	★★	★	★	★	8
Zhang 2019	★	★	★	★	★★	–	–	★	7
Elghiaty 2019	★	★	★	★	★★	–	★	★	8
Kang 2018 1	★	★	–	–	★★	★	★	★	7
Kang 2018 2	★	★	–	–	★★	★	★	★	7
Song 2019	★	★	★	★	★★	–	★	★	8
Takemura 2020	★	★	–	★	★★	–	★	★	7
Zheng 2018	★	★	–	★	★★	★	★	★	8
Ishihara 2017	★	★	–	★	★★	–	★	★	7
Xu 2018	★	★	–	★	★★	★	★	★	8
Bao 2020	★	★	★	★	★★	–	★	★	8
Suzuki 2020	★	★	–	★	★★	★	★	★	8

*1* Representativeness of the exposed cohort, *2* Selection of the non-exposed cohort, *3* Ascertainment of exposure, *4* Outcome of interest not present at start of study, *5* Control for important factor or additional factor, *6* Assessment of outcome, *7* Follow-up long enough for outcomes to occur, *8* Adequacy of follow up of cohorts

### The CONUT score and OS in urological cancers

**Fig. 2:**
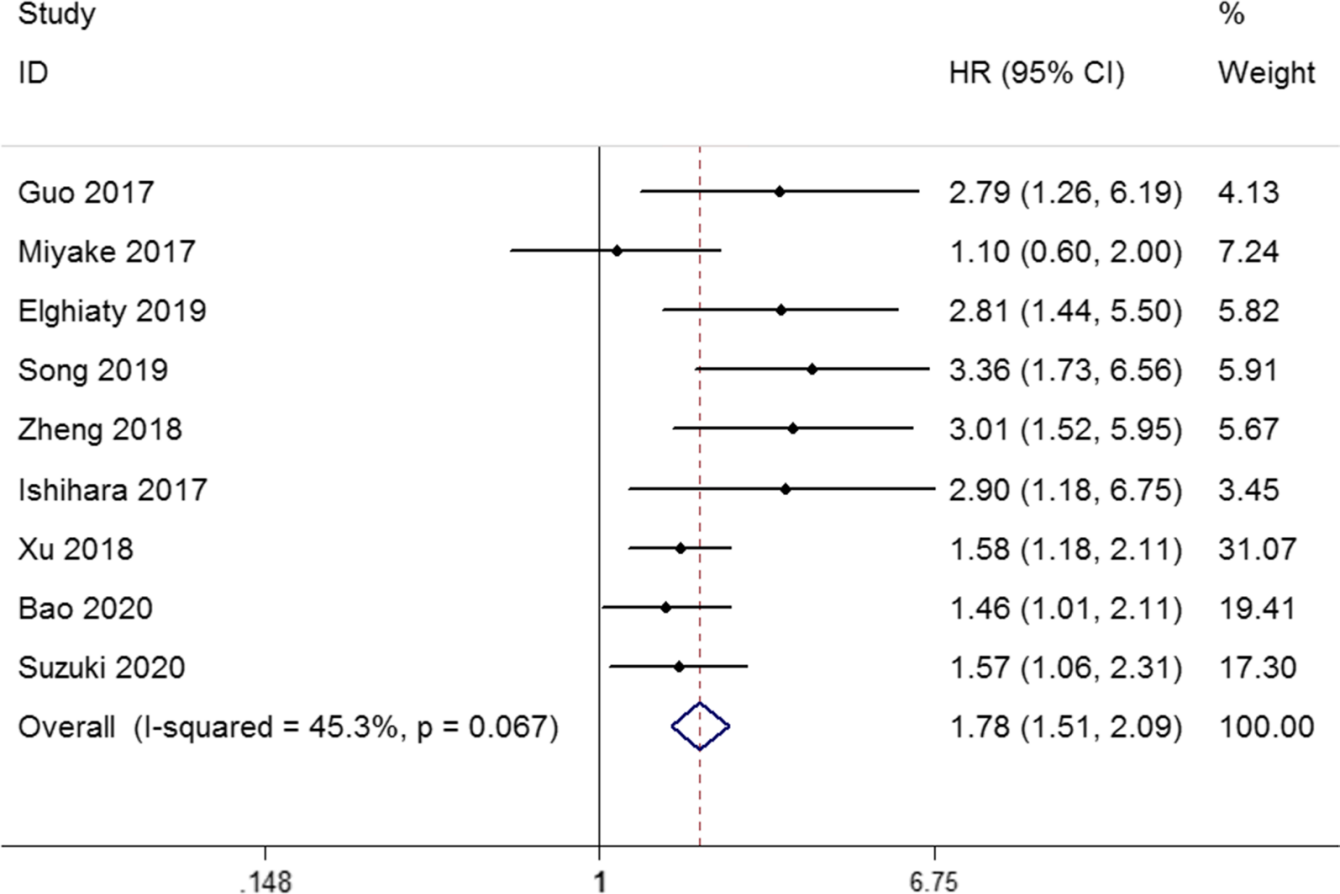
Forest plot of the relationship between high CONUT score and OS

A total of 9 studies with 3562 patients explored the role of CONUT score on OS. The fixed-effect model was used to calculated the pool results (I^2^ = 45.3%, p = 0.067). As shown in Fig. 2, the pooled data revealed that increased CONUT score was associated with worse OS (HR: 1.78, 95% CI 1.51–2.09, p < 0.001). Subgroup analysis by cancer type for OS indicated that elevated CONUT score can predict poor OS for RCC (HR: 3.05, 95% CI 2.07–4.50, p < 0.001) and UC (HR: 1.58, 95% CI 1.32–1.89, p < 0.001). After stratification by cancer stage, the pooled HR was 2.14 (95% CI 1.45–3.15, p < 0.001) in non-metastatic group and 1.69 (95% CI 1.29–2.22, p < 0.001) in mixed group. In terms of treatment methods, pretreatment CONUT score could be a negative predictor for OS in patients undergoing surgery (HR: 2.02, 95% CI 1.53–2.68, p < 0.001). In the analysis of sample size, the pooled HR was 1.73 (95% CI 1.40–2.12, p < 0.001) for sample size > 400 and 2.02 (95% CI 1.33–3.07, p < 0.001) for sample size < 400. In addition, when the cut-off value of CONUT score = 3 (HR: 2.31, 95% CI 1.44–3.72, p = 0.001) and CONUT score ≤ 2 (HR: 1.68, 95% CI 1.38–2.05, p < 0.001), it was also negatively associated with OS. Finally, among Asian populations, high CONUT score could be a negative predictor for OS in urological cancers (HR: 1.73, 95% CI 1.46–2.04, p < 0.001). These results are illustrated in Table 4.

#### The CONUT score and CSS in urological cancers

**Fig. 3:**
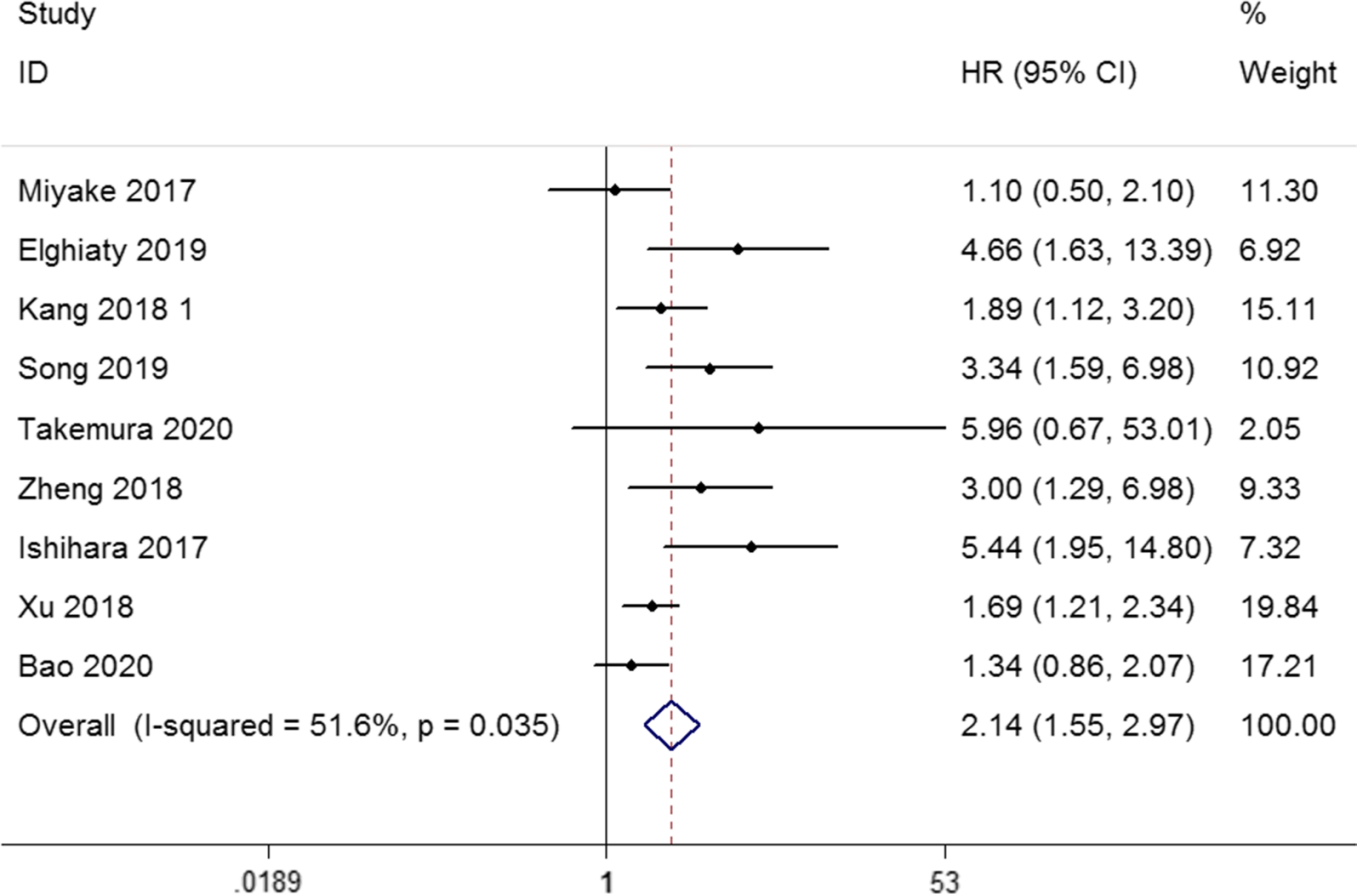
Forest plot of the relationship between high CONUT score and CSS

The effect of CONUT score on CSS was evaluated in 4677 patients from 9 studies. A combined analysis demonstrated that high CONUT score were significantly positively correlated with shortened CSS (HR: 2.14, 95% CI 1.55–2.97, p < 0.001), with moderate heterogeneity identified between studies (I^2^ = 51.6%, p = 0.035; Fig. 3). Subgroup analysis by cancer type for CSS indicated that elevated CONUT score can predict poor CSS for RCC (HR: 2.67, 95% CI 1.87–3.81, p < 0.011) and UC (HR: 1.68, 95% CI 1.09–2.59, p = 0.019). After stratification by cancer stage, the pooled HR was 2.45 (95% CI 1.44–4.17, p = 0.001) in non-metastatic group and 1.74 (95% CI 1.32–2.31, p < 0.001) in mixed group. In terms of treatment methods, preoperation CONUT score could be a negative predictor for CSS (HR: 2.10, 95% CI 1.51–2.92, p < 0.001). In the analysis of sample size, the pooled HR was 1.77 (95% CI 1.41–2.20, p < 0.001) for sample size > 400 and 2.83 (95% CI 1.23–6.50, p = 0.014) for sample size < 400. In addition, when the cut-off value of CONUT score = 3 (HR: 2.66, 95% CI 1.13–6.27, p = 0.026) and CONUT score ≤ 2  (HR: 1.85, 95% CI 1.45–2.35, p < 0.001), it was also negatively associated with CSS. Finally, CONUT score was also an appropriate biomarker to predict worse CSS among Asian populations (HR: 1.82, 95% CI 1.49–2.23, p < 0.001). These results are illustrated in Tables 4, 5.

**Table 4:** Subgroup analyses of overall survival

Subgroup	No. of studies	HR (95% CI)	P	Heterogeneity		Model
				I^2^ (%)	Ph	
Cancer type						
RCC	3	3.05 (2.07–4.50)	< 0.001	0.0	0.933	Fixed
UC	6	1.58 (1.32–1.89)	< 0.001	7.3	0.370	Fixed
Cancer stage						
Non-metastatic	6	2.14 (1.45–3.15)	< 0.001	58.1	0.036	Random
Metastatic	1	1.57 (1.06–2.31)	0.023	–	–	–
Mixed	2	1.69 (1.29–2.22)	< 0.001	42.2	0.189	Fixed
Treatment						
Surgery	8	2.02 (1.53–2.68)	< 0.001	50.6	0.048	Random
Mixed	1	1.57 (1.06–2.31)	0.023	–	–	–
Sample size						
> 400	4	1.73 (1.40–2.12)	< 0.001	47.9	0.124	Fixed
< 400	5	2.02 (1.33–3.07)	0.001	53.9	0.070	Random
Cuf-off						
3	4	2.31 (1.44–3.72)	0.001	54.0	0.089	Random
≤ 2	5	1.68 (1.38–2.05)	< 0.001	45.0	0.122	Fixed
Population						
Asian	8	1.73 (1.46–2.04)	< 0.001	44.9	0.08	Fixed
African	1	2.81 (1.44–5.50)	0.003	–	–	–

**Table 5:** Subgroup analyses of cancer-specific survival

Subgroup	NO. of studies	HR (95% CI)	P	Heterogeneity		Model
				I^2^ (%)	Ph	
Cancer type						
RCC	5	2.67 (1.87–3.81)	< 0.001	0.0	0.453	Fixed
UC	4	1.68 (1.09–2.59)	0.019	59.5	0.060	Random
Cancer stage						
Non-metastatic	6	2.45 (1.44–4.17)	0.001	66.4	0.011	Random
Metastatic	1	5.96 (0.67–53.01)	0.109	–	–	–
Mixed	2	1.74 (1.32–2.31)	< 0.001	0.0	0.721	Fixed
Treatment						
Surgery	8	2.10 (1.51–2.92)	< 0.001	54.7	0.031	Random
Target therapy	1	5.96 (0.67–53.01)	0.109	–	–	–
Sample size						
> 400	5	1.77 (1.41–2.20)	< 0.001	38.2	0.166	Fixed
< 400	4	2.83 (1.23–6.50)	0.014	64.6	0.037	Random
Cuf-off						
3	3	2.66 (1.13–6.27)	0.026	77.4	0.012	Random
≤ 2	6	1.85 (1.45–2.35)	< 0.001	34.4	0.178	Fixed
Population						
Asian	8	1.82 (1.49–2.23)	< 0.001	48.5	0.059	Fixed
African	1	4.66 (1.62–13.39)	0.004	–	–	–

##### The CONUT score and RFS/DFS/PFS in urological cancers

**Fig. 4:**
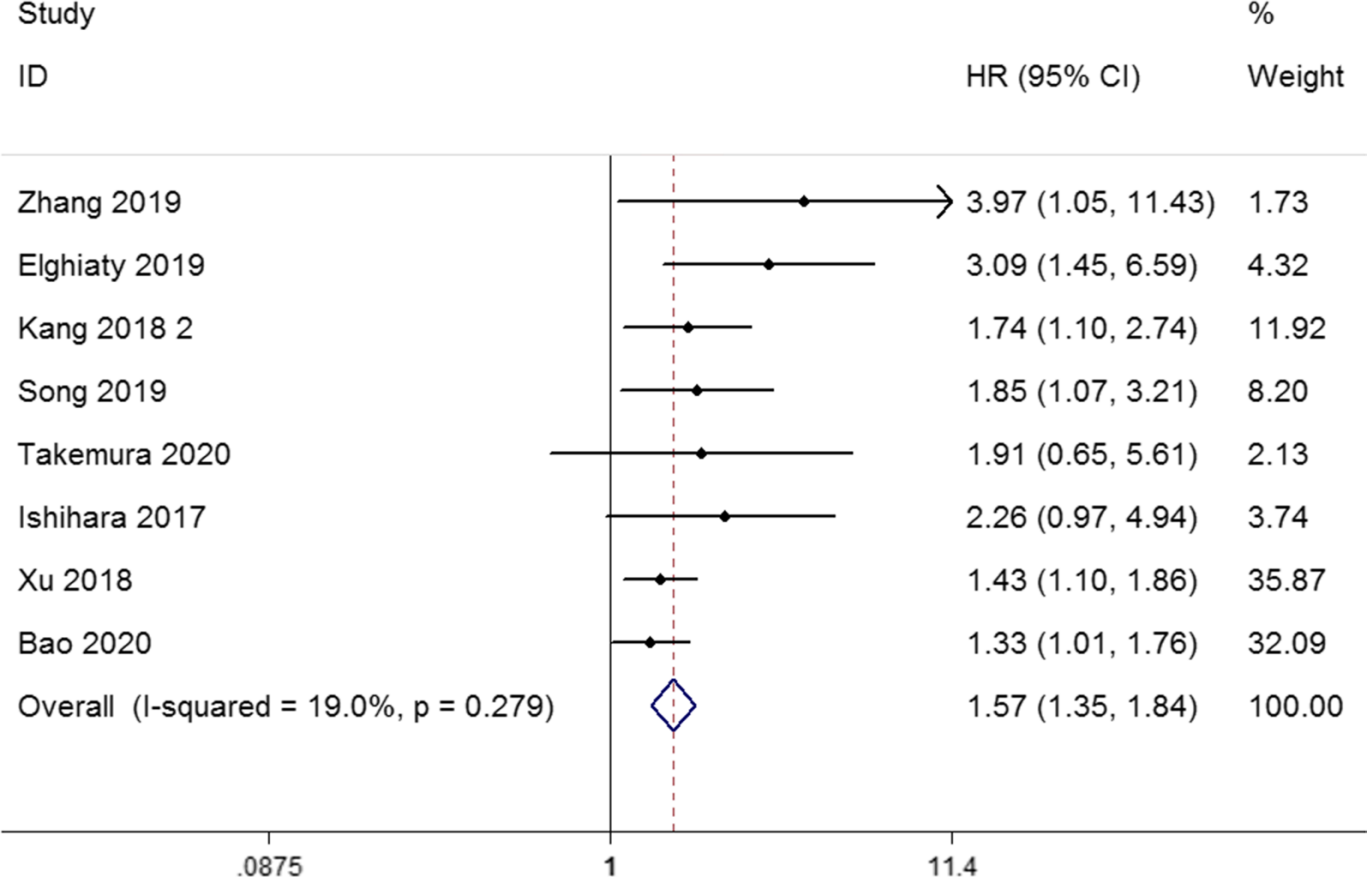
Forest plot of the relationship between high CONUT score and RFS/DFS/PFS

As shown in Fig. 4, a total of 8 eligible studies comprising 3883 patients revealed the relationship between CONUT score and RFS/DFS/PFS by fixed-effects model with low heterogeneity (I^2^ = 19.0%, p = 0.279) in urological cancers (HR: 1.57, 95% CI 1.35–1.84, p < 0.001) especially in RCC (HR: 1.96, 95% CI 1.44–2.66, p < 0.001) and UC (HR: 1.42, 95% CI 1.18–1.71, p < 0.001). Subgroup analysis by treatment methods indicated that CONUT score could be a negative predictor for RFS/DFS/PFS in patients undergoing surgery (HR: 1.57, 95% CI 1.34–1.84, p < 0.001). In terms of cancer stage the pooled HR was 1.61 (95% CI 1.32–1.97, p < 0.001) in non-metastatic group and 2.65 (95% CI 1.19–5.90, p = 0.017) in metastatic group. After stratification by sample size, the pooled HR was 1.49 (95% CI 1.25–1.77, p < 0.001) in sample size>400 group and 2.12 (95% CI 1.43–3.15, p < 0.001) in sample size<400 group. Moreover, when the cut-off value of CONUT score = 3 (HR: 1.54, 95% CI 1.22–1.94, p < 0.001) and CONUT score≤2 (HR: 1.61, 95% CI 1.30–1.99, p < 0.001), it was also negatively associated with RFS/DFS/PFS. Finally, high CONUT score was also associated with poor RFS/DFS/PFS among Asian populations (HR: 1.53, 95% CI 1.30–1.79, p < 0.001). These results are shown in Table 6.

**Table 6:** Subgroup analyses of recurrence/disease/progress-free survival

Subgroup	NO. of studies	HR (95% CI)	P	Heterogeneity		Model
				I^2^ (%)	Ph	
Cancer type						
RCC	4	1.96 (1.44–2.66)	< 0.001	0.0	0.636	Fixed
UC	3	1.42 (1.18–1.71)	< 0.001	0.0	0.483	Fixed
Cancer stage						
Non-metastatic	5	1.61 (1.32–1.97)	< 0.001	29.2	0.227	Fixed
Metastatic	2	2.65 (1.19–5.90)	0.017	0.0	0.372	Fixed
Mixed	1	1.43 (1.10–1.86)	0.008	–	–	–
Treatment						
Surgery	7	1.57 (1.34–1.84)	< 0.001	29.6	0.202	Fixed
Target therapy	1	1.91 (0.65–5.61)	0.239	–	–	–
Sample size						
> 400	4	1.49 (1.25–1.77)	< 0.001	36.5	0.193	Fixed
< 400	4	2.12 (1.43–3.15)	< 0.001	0.0	0.716	Fixed
Cuf-off						
3	4	1.54 (1.22–1.94)	< 0.001	36.6	0.192	Fixed
≤ 2	4	1.61 (1.30–1.99)	< 0.001	21.9	0.279	Fixed
Population						
Asian	7	1.53 (1.30–1.79)	< 0.001	0.0	0.487	Fixed
African	1	3.09 (1.45–6.59)	0.003	–	–	–

## Publication bias and sensitivity analyses

**Fig. 5:**
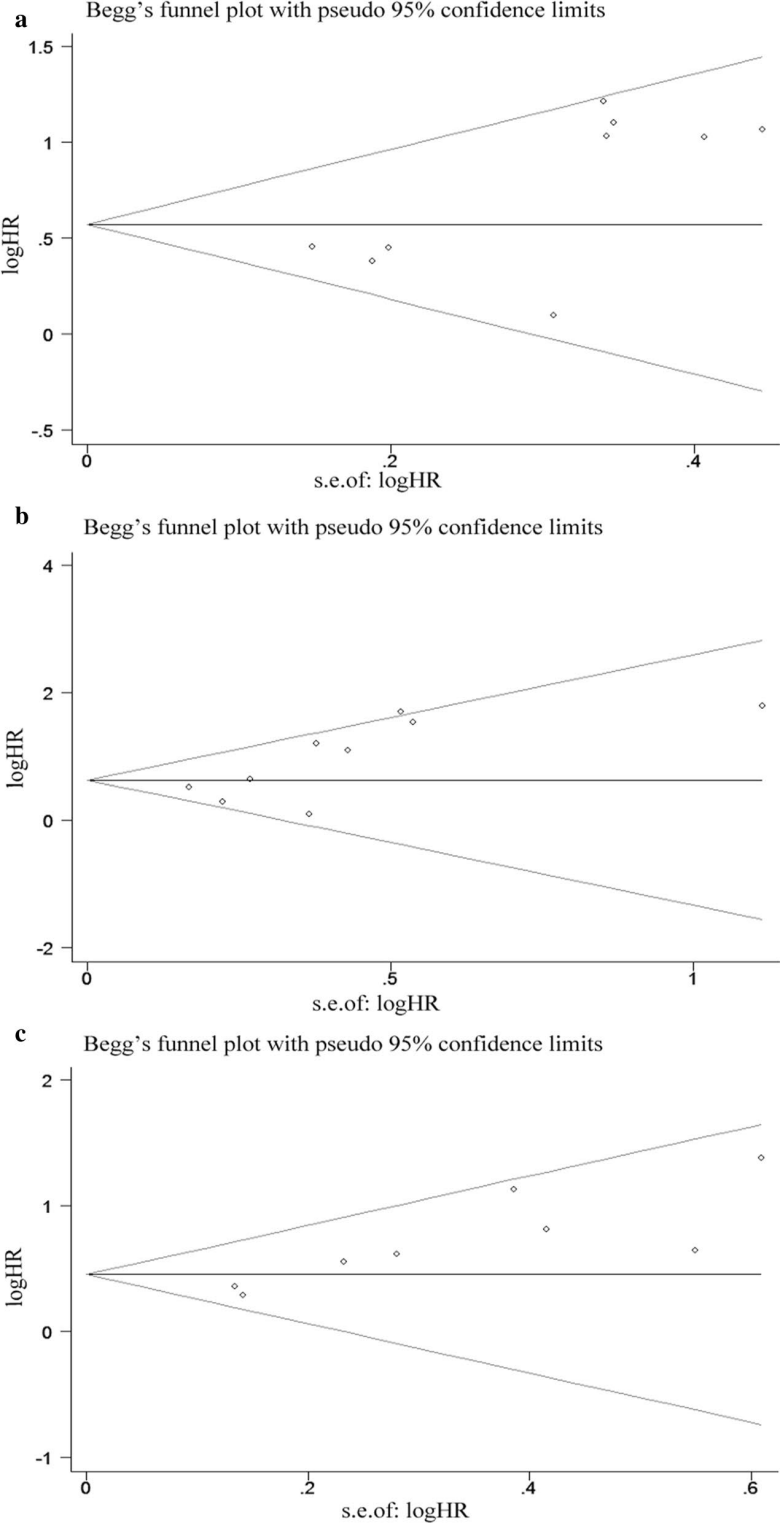
Begg’s publication bias funnel plots for the correlation of high CONUT score with OS (**a**); with CSS (**b**); with RFS/DFS/PFS (**c**)

**Fig. 6:**
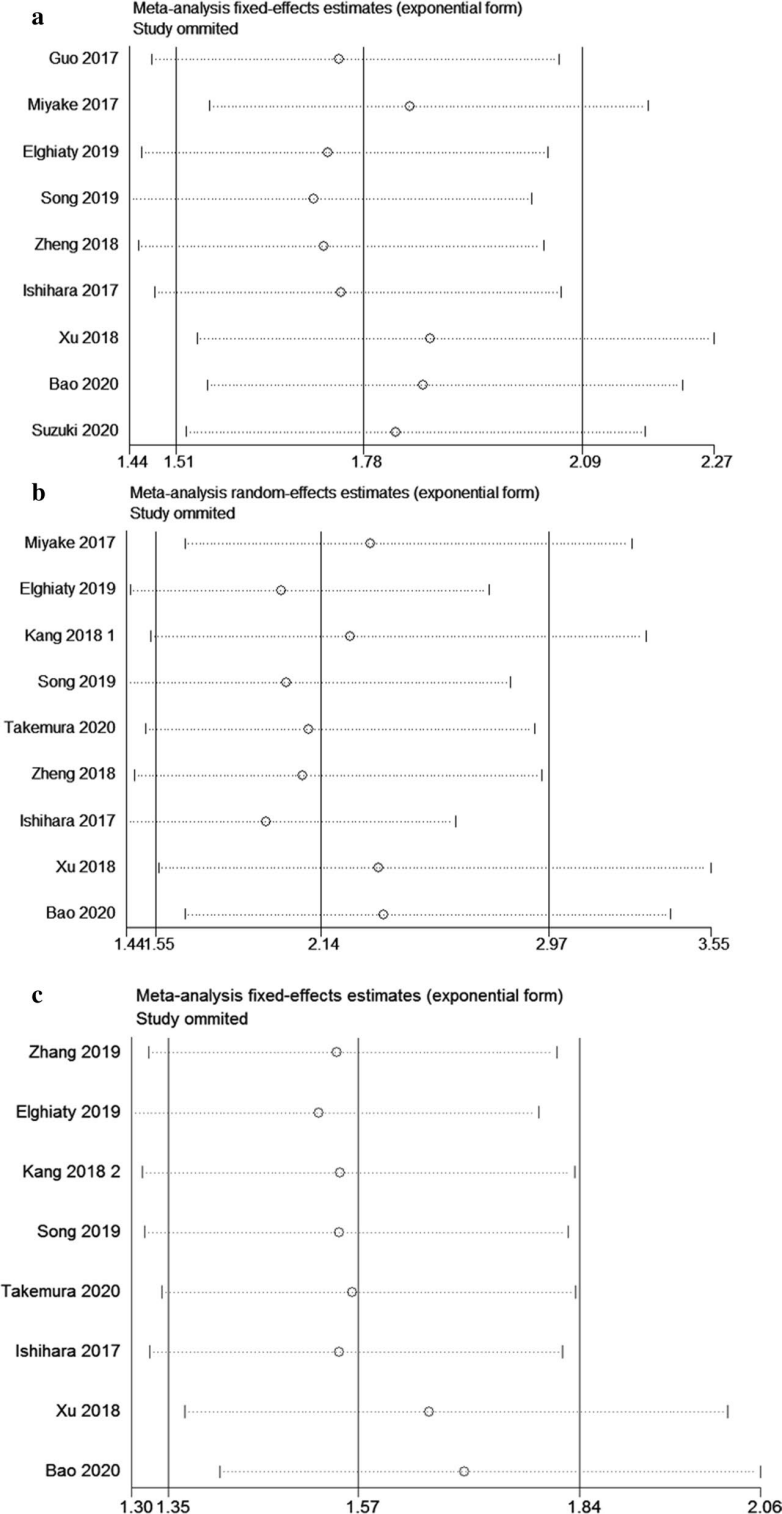
Sensitivity analysis for the correlation of high CONUT score with OS (**a**); with CSS (**b**); with RFS/DFS/PFS (**c**)

The funnel plots of Bgger’s test were displayed in Fig. 5. Bgger’s test revealed that no significant publication bias in this meta-analysis about CONUT score and OS (Fig. 5a, p = 0.348), CSS (Fig. 5b, P = 0.076) and RFS/DFS/PFS (Fig. 5c, p = 0.108). By estimating the potential impact of individual studies on the pooled data, sensitivity analysis was carried out. It was obvious that, pooled HR was not remarkably altered when each single study was removed in turn (Fig. 6a: OS, Fig. 6b: CSS, Fig. 6c: RFS/DFS/PFS).

## Discussion

 According to our best knowledge, this is the first meta-analysis which systemically investigated the relationship between CONUT score and the prognosis of urological cancers patients. This meta-analysis of 13 studies demonstrated that high CONUT score predicted poor OS, CSS and RFS/DFS/PFS in urological cancers. A stratified analysis demonstrated that high CONUT score were significantly correlated with decreased OS, CSS and RFS/DFS/PFS, irrespective of the cancer type, cancer stage, treatment methods, sample size and cut-off value. Our results gave the information that pretreatment CONUT score may represent an independent prognostic indicator in patient with urological cancers. Urological cancers contain RCC, PC and UC. RCC is the 13th most common cause of cancer-related mortality worldwide [30]. It is utmostly important to identify the prognostic factors in RCC. Our results confirmed that high COUNT score was associated with worse OS, CSS and RFS/DFS/PFS and CONUT score was an effective prognostic biomarker for RCC. UC, mostly comprising BC and UTUC, have a poor prognosis due to the distant metastasis or postoperative recurrence [31]. To investigate an appropriate prognostic marker is urgent. The subgroup analysis in this study showed that high CONUT score was a prognostic marker for worse OS in UC. Similarly, high CONUT score was also negatively correlated with CSS and RFS/DFS/PFS. Among the included studies in this meta-analysis, only one article investigated the relationship between CONUT score and PC. In that study, the researchers assessed the PFS of CONUT score in PC and considered high CONUT score could predict poor PFS in PC.

The biological mechanism regarding the association between the CONUT score and prognosis is still unclear. However, there are several reasons to explain why a high CONUT score is associated with poor outcomes in urological cancers. High CONUT score are closely related to low levels of serum albumin, cholesterol and lymphocytes. Serum albumin is a major indicator of nutritional status and has been proved associated with worse prognosis in urological cancers [11]. The decreased level of albumin correlated with the production of pro-inflammatory cytokines, such as interleukin-6, a cytokine associated with progression of cancers [32]. Cholesterol is an essential lipid for maintaining cellular, decreased level of cholesterol means a loss of cholesterol from the membrane of cells, which affects the ability of immunocompetent cells to fight against cancer cells [33, 34]. Lymphocytes can show an antitumor role through the induction of cytotoxic cell death [35], and perioperative lymphopenia was reported to be associated with inferior prognosis in cancers [36]. Thus, high CONUT score is typically associated with the invasion and metastasis of tumour cells and it can lead to poor survival.

This meta-analysis had several limitations. First, all enrolled studies were retrospective design, which might contain a potential selection bias. Second, most included studies were performed in Asian countries, therefore it remains unknown whether our results can be applied to Western populations. Third, the cut-off values were inconsistent among the studies. The cutoffs in enrolled studies didn’t reach the standard point. Although, according to CONUT = 3 in the subgroup analysis, we pooled the results, bias may exist because the use of diverse cut-off values caused different conclusions in the same article. Fourth, relevant studies were too few in some subgroup analysis such as only one study investigated the outcome of CONUT score in PC. Moreover, moderate heterogeneity existed in the analysis of CSS. Finally, high CONUT score may have been affected not only by aggressive tumor but also by old age and underlying disease. These articles did not adequately adjust for these confounding factors.

## Conclusions

Our results display that pretreatment CONUT score represents an independent predictive factor for OS, CSS and RFS/DFS/PFS, and the CONUT score may represent an effective prognostic indicator in urological cancers.

## Data Availability

The information used and analyzed during this study is available from the original literature listed in the reference. The analysis methods can be obtained from the corresponding author upon reasonable request.

## Abbreviations

CONUT: Controlling nutritional status
CNKI: National knowledge infrastructure
HR: Hazard ratio
CI: Confidence interval
BC: Bladder cancer
PC: Prostate cancer
RCC: Renal cell carcinoma
UTUC: Upper tract urinary cancer
UC: Urothelial cancer
OS: Overall survival
CSS: Cancer-specific survival
DFS: Disease-free survival
RFS: Recurrence-free survival
PFS: Progress-free survival
PNI: Prognostic nutritional index
GPS: Glasgow prognostic score
AGR: Albumin-to-globulin ratio
PRISMA: Preferred reporting items for systemic reviews and meta-analysis
NOS: Newcastle-Ottawa Scale
